# Aerosol immunization by alginate coated mycobacterium (BCG/MIP) particles provide enhanced immune response and protective efficacy than aerosol of plain mycobacterium against *M.tb. H37Rv* infection in mice

**DOI:** 10.1186/s12879-019-4157-2

**Published:** 2019-07-01

**Authors:** Puja S. Nagpal, Ashwani Kesarwani, Parul Sahu, Pramod Upadhyay

**Affiliations:** National Institute of Immunology, Aruna Asaf Ali Marg, New Delhi, 110067 India

**Keywords:** Tuberculosis, Vaccine, BCG, Aerosol immunization, Alginate coated mycobacterium

## Abstract

**Background:**

With the aim of preparing a more effective, safe and economical vaccine for tuberculosis, inhalable live mycobacterium formulations were evaluated.

**Methods:**

Alginate particles in the size range of 2–4 μm were prepared by encapsulating live Bacille Calmette–Guérin (BCG) and “*Mycobacterium indicus pranii”* (MIP). These particles were characterized for their size, stability and release profile. Mice were immunized with liquid aerosol or dry powder aerosol (DPA) alginate encapsulated mycobacterium particles and their in-vitro recall response and infection with mycobacterium H37Rv were investigated.

**Results:**

It was found that the DPA of alginate encapsulated mycobacterium particles invoked superior immune response and provided higher protection in mice than the liquid aerosol. The BCG encapsulated in alginate particles (BEAP) and MIP encapsulated in alginate particles (MEAP) were engulfed by bone marrow dendritic cells (BMDCs) and co-localized with lysosome. The MEAP/BEAP activated BMDCs exhibited higher chemotaxis movement and had enhanced ability of antigen presentation to T cells.

The in-vitro recall response of BEAP/MEAP immunized mice when compared in terms of proliferation index and Interferon gamma (IFN-gamma) released by splenocytes and mediastinal lymph node cells was found to be higher than mice immunized by liquid aerosol of BCG/MIP. Finally, different groups of immunized mice were infected with *M. tb* H37Rv and after 16 weeks the Colony forming units (CFUs) in lung and spleen estimated. The bacilli burden in the BEAP/MEAP immunized mice was significantly less than the respective liquid aerosol immunized mice and the histopathology of BEAP/MEAP immunized mice lungs showed very little damage.

**Conclusions:**

These inhale-able vaccines formulation of alginate coated live mycobacterium are more immunogenic as compared to the aerosol of bacilli and they provide better protection in mice when infected with H37Rv.

**Electronic supplementary material:**

The online version of this article (10.1186/s12879-019-4157-2) contains supplementary material, which is available to authorized users.

## Background

There is unmet need for better vaccine technologies for the treatment and prevention of infectious diseases. Tuberculosis (TB) is one of the major infectious diseases which accounts for very high morbidity and mortality around the world. Bacille Calmette–Guérin (BCG), the only vaccine available against tuberculosis has variable efficacy [1]. There are a few candidate TB vaccines in the pipeline [2–8] and there have been many attempts to improve the efficacy of existing TB vaccines by investigating alternate mode of delivery [9, 10] and formulations [11].

The pulmonary route of immunization has been shown to generate more protective immune response in lung diseases because “immune responses are often strongest in compartments proximal to the site of vaccine application” [12]. The pulmonary delivery can be achieved by liquid aerosol or by dry powder aerosol (DPA). DPA has been preferred in many formulations of therapeutic drugs [13–17] and vaccines [18, 19] as it is easy to generate and has higher stability at room temperature. Moreover, the pulmonary delivery being a needle free immunization method has many obvious advantages like safety, ease of delivery etc. [20, 21].

Since TB is mainly a lung disease, a vaccine against TB delivered directly to lung is expected to provide higher protection as compared to any other route. There have been many reports which support this hypothesis [22, 23]. In order to achieve longer shelf life and ease of delivery, Lucila Garcia-Contreras et al. [24, 25] prepared DPA of BCG by spray drying.

In a few vaccine formulations, the encapsulation of antigen in the polymeric particles is known to provide adjuvant like effect which enhances their protective efficacy [26–30]. We encapsulated BCG and Mycobacterium indicus pranii (MIP) in bio-polymeric alginate micro-particles, formulated the encapsulated mycobacterium as DPA and investigated the improvement obtained in terms of their immunogenicity and protective efficacy.

## Methods

### Animals

All animal experiments were carried out on inbred, 4–6 weeks old C57BL/6 or Balb/c mice obtained from Jackson’s laboratory, USA and bred and maintained in the small animal facility of the National Institute of Immunology, New Delhi. The animals received ad libitum access to acidified autoclaved water and food. The temperature and humidity of the housing room was maintained at 21–23 °C and 40–60% respectively. Animals were kept at a 14 h light - 10 h dark cycle. Typically, four animals were randomly assigned in each group and the entire experiment was repeated twice. All animal experiments and reporting adhere to the ARRIVE guidelines [31].

### Reagents

D(+) Trehalose dihydrate, Calcium chloride, Polyvinyl alcohol (M.W. 30,000 - 70,000) was purchased from Sigma Aldrich (USA). Sodium salt of Alginic acid (brown algae) was procured from Fluka Biochemika (USA). For all experiments MilliQ water was used. Cells for in vitro experiments were maintained in RPMI media (HiMedia, India) supplemented with 10% v/v fetal bovine serum (FBS) (HiMedia, India) and 1% antibiotic solution procured from Sigma Aldrich (USA). All other tissue culture reagents and chemicals required for buffer were purchased from Sigma–Aldrich (USA). Mycobacterium cultures were grown in either Difco Middlebrook 7H9 liquid or 7H11 solid Media (BD biosciences, USA) supplemented with 10% ADC or OADC solution (BD Biosciences, USA). For long term storage, cultures were maintained on LJ media (BD Bioscience, USA). Hygromycin antibiotic required for GFP mycobacterium was procured from Himedia, India. Danish 1331 strain Bacillus Calmette-Guérin (BCG) and an “in-house” maintained strain of “*Mycobacterium indicus pranii”* (MIP) were used. GMCSF and CCL21 (Murine Exodus-2) protein was obtained from Peprotech. Commercially available LDH and IgE estimation kit were purchased from “EIAab & USCNLIFE” and “Immunology Consultants Laboratory” respectively. All other Enzyme linked immunosorbent assay (ELISA) kits and antibodies were purchased from BD biosciences or e-bioscience USA.

### Preparation of viable mycobacterium encapsulated alginate particles

Mycobacterium (MIP/BCG) cultures were grown in 7H9 media supplemented with 10% ADC in a shaking incubator at 150 rpm at 37 °C. At optical density 0.9, culture was harvested by centrifugation at 1000 g for 10 min. Bacterial pellet obtained was washed twice with phosphate buffer saline (PBS) pH 7.2. Pellet equivalent to 10^10^ bacilli was re-suspended in 50 ml solution containing 1.23% sodium alginate and 8.25% of trehalose. 5 ml of this suspension was filled in a laboratory made nebulization assembly and nebulized with a piston based air pump to generate aerosol of sodium alginate and MIP/BCG. A schematic diagram of nebulization assembly is shown in Additional file 1: Figure S1. The generated aerosol was entrapped in 5% solution of calcium chloride containing 0.1% polyvinyl alcohol with constant stirring. After 12 h of the nebulization, gelled particles were collected by spinning at 350 g for 10 min. Particles were then washed three times with MilliQ water to completely remove residual calcium chloride. Finally, the pellet was re-suspended in 5 ml MilliQ water, followed by snap freezing in liquid nitrogen and dried in a lyophilizer.

### Stability

In order to perform the accelerated stability testing the formulation was stored at 40 °C ± 2 °C for 6 months and at different time points the viability of the BCG was checked by dissolving it in 1 ml 1x PBS and plating on Middlebrook 7H11 agar plates. Similarly, for cold storage stability testing which is a typical storage condition, the formulation was stored at 4 °C and at predetermined time points the formulation was dissolved in 1 ml 1x PBS and plated on Middlebrook 7H11 agar plates.

Freeze thaw stability testing helps to determine the stability of the formulation under various conditions. Formulation was put under series of temperature changes. This temperature fluctuation (TF) may mimic the change in temperature during the normal handling and transportation procedure. In this procedure the formulation was exposed to freezing temperature of -10 °C for 24 h and then allowed to thaw at room temperature for 24 h. Next, the formulation was placed at a higher temperature of 45 °C for 24 h, followed by keeping at room temperature for 24 h. This series of procedure was repeated 3 times and then the formulation was dissolved in 1 ml 1x PBS and plated on Middlebrook 7H11 agar plates and incubated at 37 °C for 2-3 weeks.

### Dry powder delivery directly to lungs of mice

Formulation was delivered directly to the lungs of 6–8 weeks old C57BL/6 mice by endotracheal intubations. Detailed procedure is summarized in Additional file section.

### Immunization

Mice were immunized twice at an interval of 15 days either with liquid aerosol or by dry powder aerosol. For liquid aerosol immunization, around 2000–3000 bacilli of MIP/BCG were established per mice by using a laboratory made aerosol chamber [32]. For dry powder aerosol immunization, 1 mg DPA formulation of BEAP/MEAP containing 2000–3000 bacilli was delivered directly to the lungs (Additional file 1: Figure S2) At predetermined time points mice lung was excised and fixed in 10% formalin. Lung was embedded in paraffin and 5 μm section was cut and stained for histology with hematoxylin and eosin (H and E). Granulomatous lesions were identified by randomly selecting 10 fields from 2 sections in each group.

### Dendritic cell culture

Bone marrow dendritic cells (BMDCs) were generated by in vitro culture of bone marrow cells from C57BL/6 mice in RPMI supplemented with 10 ng/ml Granulocyte monocyte colony stimulating factor (GM-CSF) [33]. A published procedure [34] was modified to isolate lung DCs. Standardized protocols were followed for the staining for flow cytometry, allogenic mixed lymphocyte reaction (Allo-MLR), proliferation assay and ELISA. Details are given in Additional file 1.

### Transwell migration assay

1 million Bone marrow dendritic cells (BMDCs) per well were plated in a 6 wells plate. They were treated with either 10^6^ MIP or 10^6^ BCG or 50 μg MEAP or 50 μg BEAP or 50 μg Blank alginate particles (BAP) suspended in 100 μl of PBS and incubated at 37 °C in 5% CO_2_ for 24 h. After 24 h, activated BMDCs were harvested by spinning at 300 g for 5 min. The cell pellet was re-suspended in 666 μl of RPMI media having 10% FBS and 1% antibiotics. 100 μl of this cell suspension was carefully poured in triplicates, on the 5 μm pore sized transwell chamber. In the lower chamber of transwell plate, either 600 μl of PBS or 600 μl of CCL21 (25 μg) was placed. BMDCs loaded transwell was again incubated at 37 °C in 5% CO_2_ for 1 h. After 1 h, BMDCs migrated to the lower chamber were harvested and fixed with 4% paraformaldehyde solution. BMDCs were then stained with propidium iodide after permeabalizing them with 0.1% saponin solution. The cell counts of each of the wells were estimated by flowcytometry (Cyflow, Partec).

### Fluorescent microscopy imaging

To microscopically visualize the uptake of particles by BMDCs, green fluorescent protein (gfp) expressing MIP or BCG or their corresponding MEAP/BEAP were used. Green fluorescent protein (gfp) expressing MIP and BCG were used to microscopically visualize the uptake of particles by BMDCs. Similar to other experiments; BMDCs were incubated with gfp expressing either of MEAP, BEAP, MIP or BCG baclli for 48 h. BMDCs were then washed and stained for CD11c with Alexa fluor 498 and mounted with vector mount DAPI.

### Infection

Three weeks after second dose of immunization, low dose of H37Rv aerosol infection was given to two groups of all the immunized and age matched control animals. Mice were exposed to the aerosol generated by Glass-Col inhalation exposure system to establish 200–400 bacilli of H37Rv per mouse [35].

### Estimation of colony forming units (CFU) load in lung and spleen

Mice were euthanized at 4 and 16 weeks post infection by intraperitoneal injection (0.1 ml/10 g body weight) of a mixture consisting high dose of anesthetic drugs, Ketamine (100 mg/ml) and Xylazine (10 mg/ml). Lungs and spleen were removed, washed and homogenized in 1 ml 7H9 media. Homogenates were evaluated for bacilli load by plating different dilution in triplicate on LJ plates having MGI PANTA antibiotic mixture (BD, USA). After 3-4 weeks of incubation at 37 °C plates were examined for CFUs. Weights of another group of mice infected with *M.tb. H37Rv* were monitored at regular intervals of time.

In every H37Rv aerosol infection procedure, typically 3 animals from un-immunized (control) group were euthanized on day 1 and number of delivered bacilli to lungs were estimated by plating lungs homogenate.

### Statistical analysis

Statistical analysis on data was done by using the GraphPad Prism, version 7, program. All the data were plotted and calculated as the mean ± standard deviation (SD). The comparison was made among the groups by analysis of variance (ANOVA) and Tukey’s correction. ‘p’ value less than 0.05 was considered significant.

## Results

### Characterization of particles

#### Size analysis and morphology of the particles

**Fig. 1:**
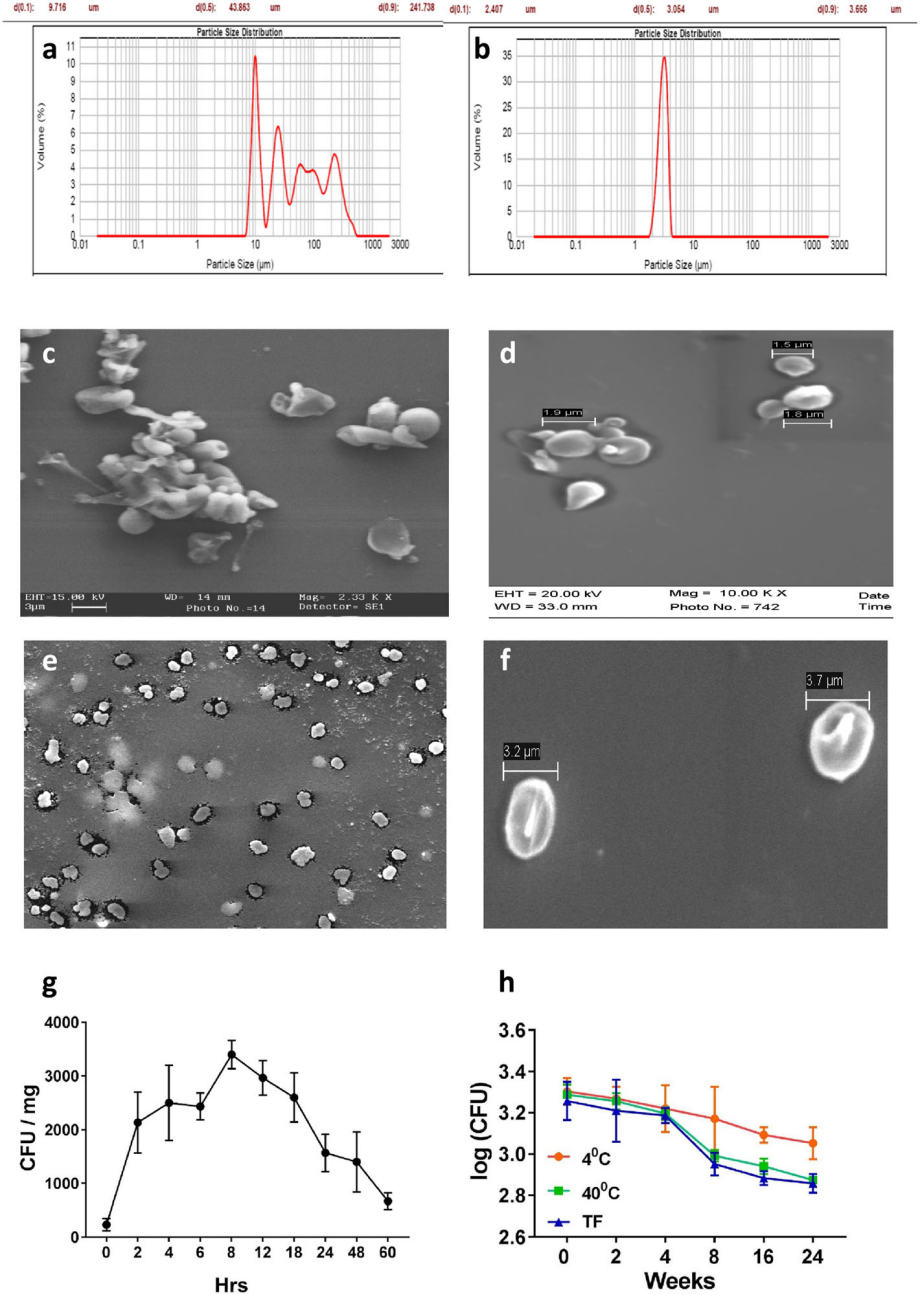
Size and SEM images of mycobacterium encapsulated alginate particles. **a** and **b** are the size profile after drying and sonication respectively. **c** and **d** show the SEM images of particle aggregates obtained after drying and images of segregated particles are shown in (**e** and **f**). The accumulated release of bacteria from a typical formulation is shown in (**g**). The entire load of bacteria was released within a few hours. **h** depicts the stability of formulation with time at three different temperature conditions; 4 °C, 40 °C and temperature fluctuation (TF). Very little viability was lost in 6 months

*Mycobacterium sp.* (BCG/MIP) encapsulated particles were prepared by gelation of alginate in calcium chloride solution. For the preparation of these particles, *Mycobacterium sp.* were suspended in the alginate and trehalose solution and aerosolized droplets of this mixture were collected in calcium chloride solution for gelling in a specially designed assembly (Additional file 1: Figure S1). The particles so formed were dried in a lyophilizer and analyzed for their size. It was found that the mass median diameter of ethanol dispersed formulation was 43 μm prior to jet milling (Fig. 1a) and size was decreased to approximately 3.5 μm when sonication was applied for 30 min. This suggested that particles might have form aggregates in the process of drying. It was confirmed by scanning electron microscopy (SEM) images (Fig. 1c and d) where aggregates of 10–20 particles were seen. After jet milling, most of the aggregates got segregated to individual particles as confirmed by SEM images (Fig. 1e and f).

#### Release kinetics of bacteria from alginate particles

It was important to find out in how much time Bacteria were released from the formulations. After drying, formulations were subjected to dissolution in PBS to study the release profile of the bacilli. Number of bacteria released were maximum at 8 h (Fig. 1g) suggesting that particles were completely disintegrated with in this time and entire load of bacteria released. Some decrease in CFU after 8 h may be due to the death of starving bacteria.

#### Encapsulation efficiency and viable load in the particles

The encapsulation efficiency was estimated by disintegrating particles in PBS prior to lyophilization. The viable bacterial load in pooled lyophilized particles was in between 4.1 × 10^4^ CFU/mg to 2.3 × 10^4^ CFU/mg of dry powder which reduced to 2.1 × 10^3^ CFU/mg to 1.7 × 10^3^ CFU/mg after jet milling.

#### Stability of the particles

One of the key advantages of alginate encapsulation was longer shelf life at room temperature. In order to check the stability of the particle we performed accelerated stability test, freeze thaw stability test and cold storage stability test. We found that formulations were quite stable up to 6 months at 40 °C (accelerated stability test) in which the viability decreased from 1.9 × 10^3^ ± 200 CFU/mg to 0.77 × 10^3^ ± 50 CFU/mg. In the case of freeze thaw stability testing after 6 months the viability reduced from 1.8 × 10^3^ ± 375 CFU/mg to 0.72 × 10^3^ ± 80 CFU/mg, similarly in the case of cold storage stability testing the viability reduced from 2.0 × 10^3^ ± 250 CFU/mg to 1.1 × 10^3^ ± 200 CFU/mg after 6 months of storage.

#### Delivery of the particles to lung

Specially designed equipment and techniques were used to deliver particles to lungs. In a typical experiment, 0.9 ± 0.1 mg of the particles corresponding to 2039 ± 226 CFU were delivered to the lungs of mice.

### In vitro analysis of the formulations

#### Activation of DCs and proliferation of T cells

**Fig. 2:**
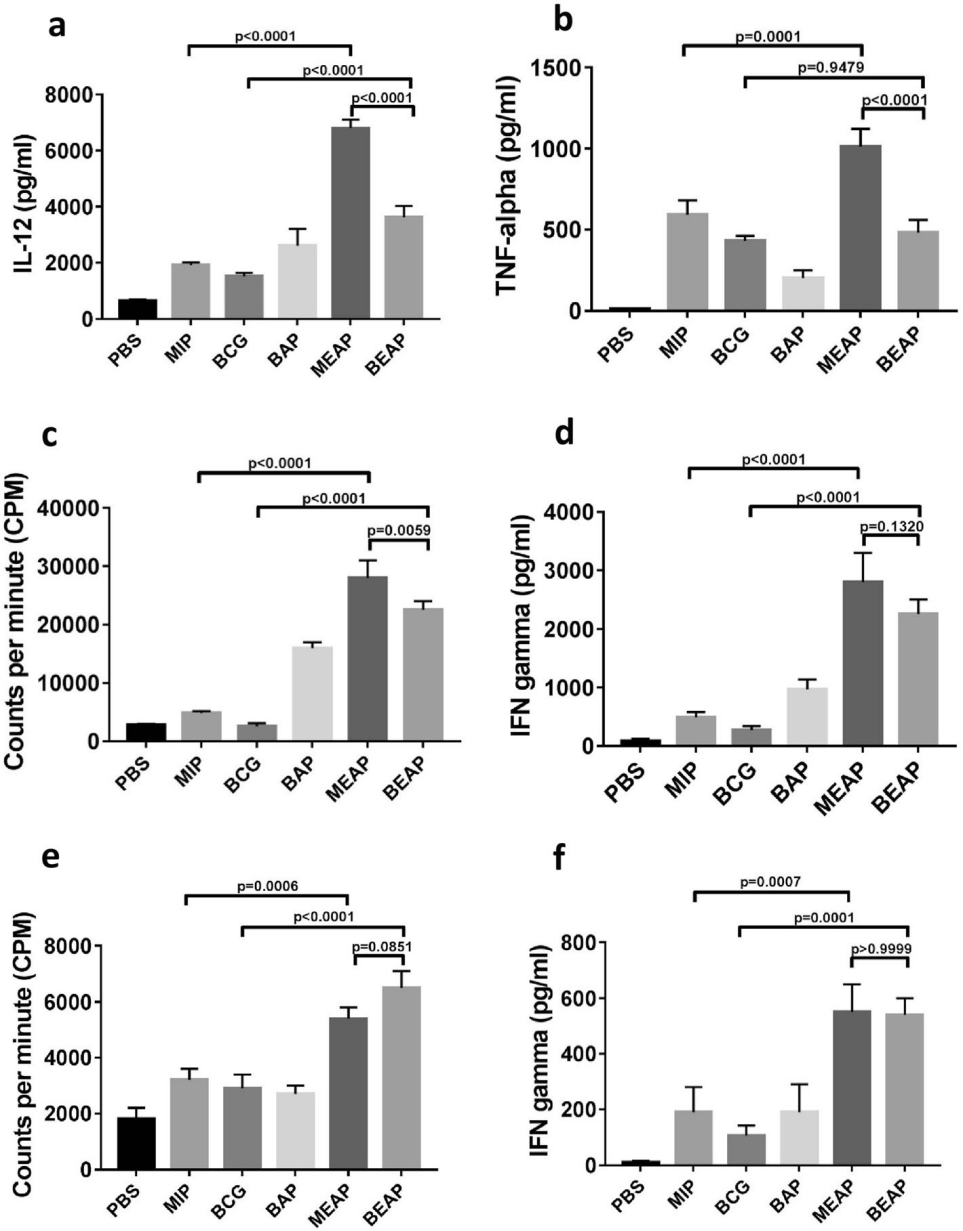
In vitro activation of DCs by different formulations. **a** and **b** show the amount of IL-12 and TNF-alpha secreted by BMDCs after 48 h of incubation with PBS, MIP, BCG, BAP, MEAP and BEAP. IFN-gamma secretion and proliferation of T cells in allogenic MLR when co-cultured with BMDCs pre-stimulated with different formulations are shown in (**c** and **d**). Similar allogenic MLR was performed with lung DCs and results are shown in (**e** and **f**). All the panels indicate that alginate coated mycobacterium leads to higher activation of DCs compared to the suspension of bacteria

To investigate the effect of the encapsulation of mycobacterium we performed a number of in vitro experiments on the bone marrow derived dendritic cells (BMDCs). As shown in the Fig. 2a, we observed higher secretion of IL-12 from BMDCs when treated with MEAP (6851 ± 11 pg) and BEAP (3721 ± 20 pg) as compared to MIP (1898 ± 68 pg), BCG (1528 ± 40 pg) and BAP (2625 ± 48 pg). Similar observation was there for the TNF-alpha release (Fig. 2b). These levels suggest that maximum activation of DCs occur when they were incubated with MEAP, followed by BEAP, MIP and BCG. Noticeable activation of DCs was also caused with blank alginate particles.

In an allogenic MLR (Fig. 2c) BMDCs treated with MEAP show very high proliferation (29,212 ± 987 cpm) of allogenic T-cells. This response is around 7.5 times higher than the control. The Interferon gamma (IFN-gamma) secretion in the supernatant of allogenic MLR also followed similar trend as shown in Fig. 2d. Similar experiments were performed with lung DCs (Fig. 2e and f) where lung DCs activated with MEAP and BEAP showed significantly higher proliferation of allogenic T cells when compared with MIP or BCG.

#### Flow cytometric analysis

We compared different activation markers like CD80, CD86, MHCII and homing receptor CCR7 on BMDCs after incubating them with BAP, BEAP/MEAP, BCG/MIP. Results are summarized in Table 1. Although some degree of activation was observed when BMDCs were incubated with BAP, the activation manifested by MIP and BCG was higher and MEAP and BEAP showed a remarkable increase of all the activation markers. These data were obtained in a representative experiment. Since different sets of BMDCs culture initiated from different animals, displayed very different expression levels in the control or basal condition itself, averaging the results obtained with different sets BMDCs culture was difficult.’

**Table 1:** Summary of a typical flow cytometry data

Groups	Percent Expression			
	CD80	CD86	MHCII - High	CCR7
Control	28.8	51.1	27.8	4.2
BAP	51.4	52.2	36.6	8
MIP	60.7	61.1	42.1	7.4
BCG	60.4	55.6	39.4	7.3
MEAP	68.3	66.3	55.3	15.6
BEAP	62.4	65.7	46.0	15.4

#### Uptake, internalization and co-localization of particle by DCs

Fluorescent microscopy images revealed that most of the micro particles were efficiently taken up by the BMDCs and maximum uptake was observed at around 24 h. In many cells more than one particle were seen inside the BMDCs. The optical sectioning of the cells confirmed that micro particles were indeed inside the cells.

**Fig. 3:**
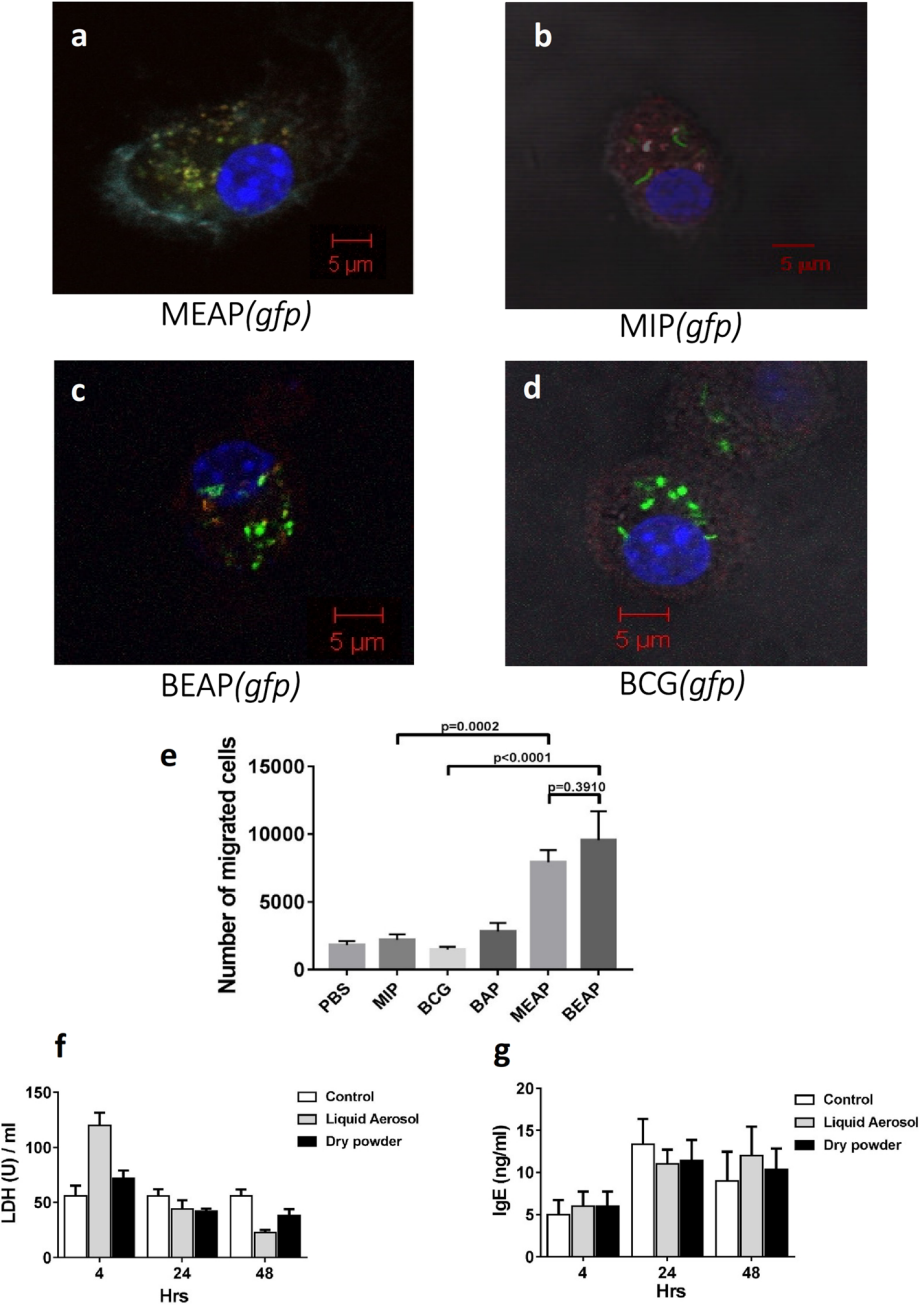
Fluorescent images showing the interaction between BMDCs and GFP-MIP, GFP-MIP encapsulated alginate micro-particles. **a**-**d** shows the co-localization of micro particles with the red stained lysosome. A representative cell after staining with different dyes was selected at random and pictures were taken in the four filter mode. The DCs after having phagocytosed MEAP(*gfp*) localized with lysosomes (red) appeared yellow (**a**). The CD11c was stained cyan and nucleus with DAPI (blue). **b**, **c** and **d** show MIP(*gfp*, BEAP*(gfp)* and BCG*(gfp)* respectively. **e** summarizes migration abilities of MEAP, BEAP, MIP, BCG and BAP activated BMDCs under the chemo-tactic influence of CCL21. 1 million BMDCs per well were plated in a 6 wells plate. They were treated with either 10^6^ MIP/BCG or 50 μg MEAP/BEAP or 50 μg BAP suspended in PBS and incubated at 37 °C in 5% CO_2_ for 24 h. After 24 h, activated BMDCs were re-suspended in RPMI media and placed on the 5 μm pore sized transwell chamber. In the lower chamber of transwell plate, either PBS or CCL21 was placed. After 1 h, BMDCs migrated to the lower chamber were estimated by flowcytometry. **f** and **g** show comparison of LDH and IgE levels after delivering liquid aerosol and dry powder aerosol at different time points in the serum of mice

The co-localization of MEAP particle within the endo-lysosomal compartments was visible as yellow (Fig. 3a) due to the overlapping of green ‘gfp MEAP’ and red stained vesicles. Further, such co-localization was less evident with MIP (Fig. 3b). Similar images were seen with BEAP and BCG (Fig. 3c and d).

#### Migration capabilities of BMDCs

One of the most obvious manifestations of the activation of DCs is their ability to migrate to the nearest lymph node after taking up the antigen. We compared the migration abilities of BMDCs in response to CCL21 after they were activated by BAP, BCG, BEAP, MIP and MEAP. The BMDCs were incubated either with BAP, BCG, MIP, BEAP or MEAP and after 48 h the chemotaxis of activated BMDCs to CCL21 was measured by the transwell assay. The MEAP/BEAP activated BMDCs showed highest migration of cells. There was not much change in the migration capacity of BMDCs when they were incubated with mycobacterium only. These results are summarized in Fig. 3e.

#### Injury to the lungs and allergic response

Mice undergo a lot of stress during the procedure of aerosol delivery and there were concerns if our specialized procedure causes any injury to the lungs. LDH levels in bronchoalveolar lavage were used to compare the injury caused by our specialized procedure and normal liquid aerosol delivery. Figure 3f shows that the LDH levels remain unchanged till 48 h when mice were immunized by liquid aerosol or dry powder indicating that ‘no’ injury was caused to the animal by the immunization procedures. Similarly, minimal allergic response (IgE) was observed (Fig. 3g) in both the groups mentioned above.

### In vivo evaluation of immune response generated by different formulations

#### Immune response in mediastinal lymph node and spleen

**Fig. 4:**
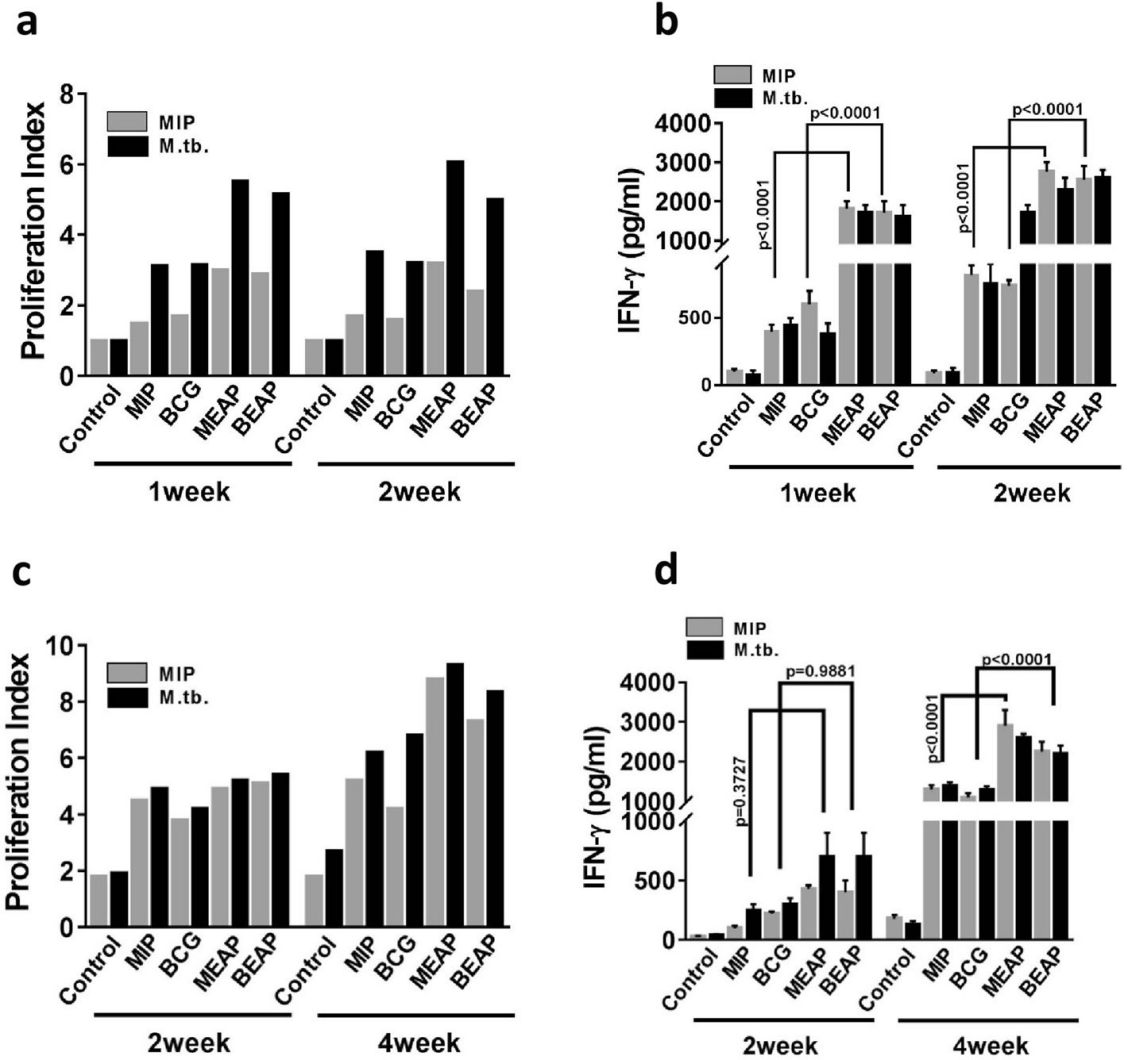
In vivo evaluations of different formulations. **a** and **b** show proliferation index and IFN-gamma secretion when mediastinal lymph node cells of animals immunized with different formulations were re-stimulated with *M.tb* and MIP antigens. Similar experiments were performed with splenocytes and results are shown in (**c** and **d**). All the panels reinforce the observation that alginate coated mycobacterium generated more pronounced immune response compared to the aerosol of bacteria

In immunized animals, the proliferation of splenocytes or T cell after antigen stimulation is an indication of antigen specific immune response. Thus, the splenocytes and mediastinal lymph node cells were isolated from the mice immunized with different formulations and were stimulated with *Mycobacterium tuberculosis (M. tb)* and MIP antigens. Figure 4 summarizes the results of proliferation and IFN-gamma secretion when cells from mediastinal lymph node (Fig. 4a and b) and splenocytes (Fig. 4c and d) were stimulated with *M.tb* and MIP antigens. In both the organs, significantly high proliferation and IFN-gamma secretion was observed in the MIP and BCG groups when compared with control. It was interesting to note that the response of MEAP and BEAP was significantly higher than the MIP and BCG. Both the antigen stimulants (MIP and *M. tb*) produced identical response.

#### Protection against *M. tb* H37Rv

The success of any vaccine ultimately depends on its ability to confer protection against actual biological infection. Thus to determine the value of alginate coated mycobacterium’s aerosol as a vaccine, we wanted to evaluate its protective efficacy. After having established the equivalence of BCG and MIP by a number of in vitro and in vivo experiments, the infection experiments were performed on MIP, BCG, BEAP and MEAP immunized animals.

**Fig. 5:**
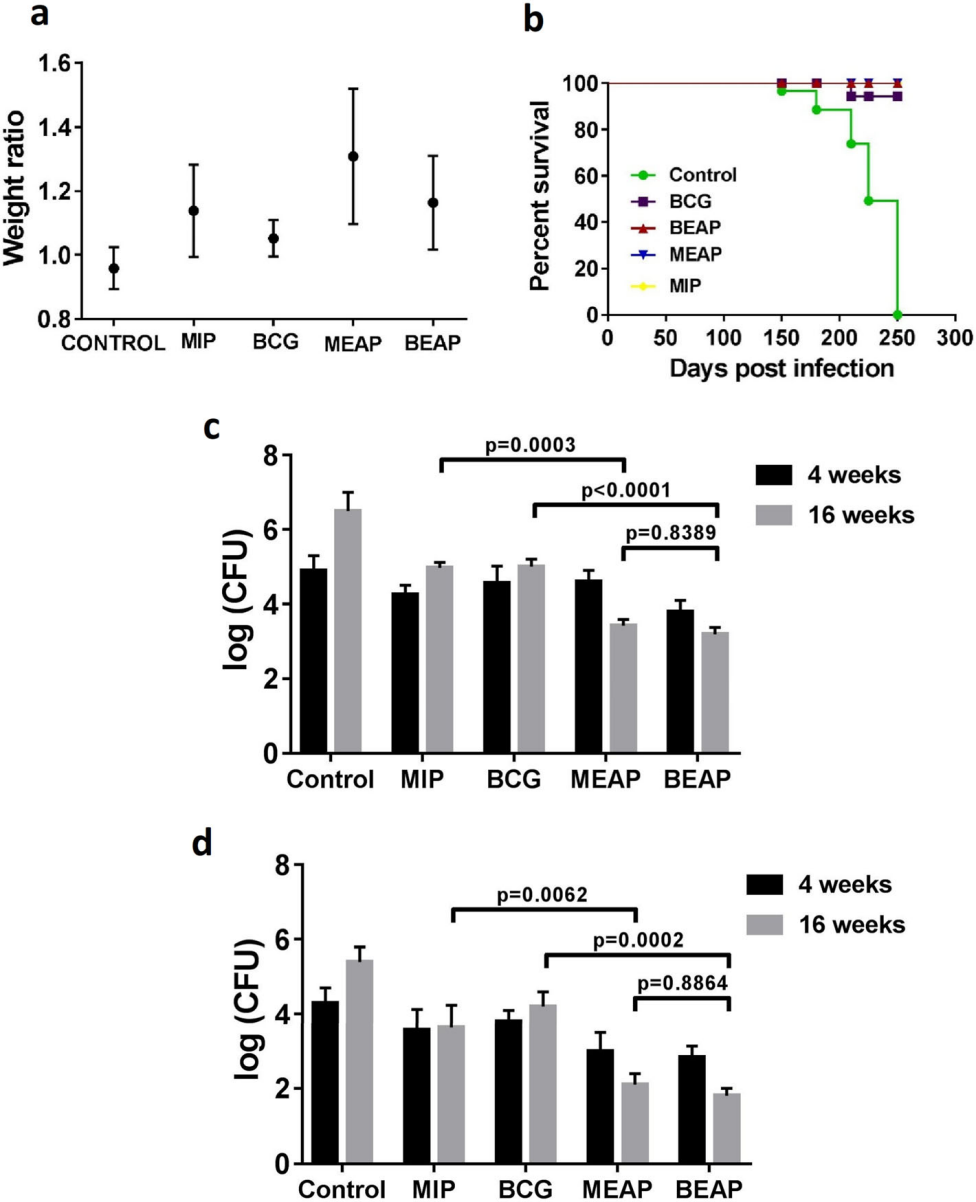
Summary of results when animals immunized with different formulations were infected with *M. tb* H37Rv. In **a**, variations in the weight ratios at 130 days post infection are shown. There was some additional gain in the weight of MEAP/BEAP immunized animals. **b** shows the survival in different groups. Immunization by all the formulations leads to nearly 100% survival. The mycobacterium load, shown as log (cfu), in the lung and the spleen of animals at 16 weeks post infection among different groups are shown in (**c** and **d**)

In the infection experiments, a low dose aerosol infection of *M. tb* (H37Rv), typically 362 ± 18 bacilli, were delivered to the mice immunized with *Mycobacterium sp.* (BCG or MIP) or their respective alginate particles (BEAP or MEAP) and their body weight and survival was observed. As shown in Fig. 5a, the body weights of animals in both the groups increased with time.

The average body weight gain at 130 days post infection was highest in the MEAP group followed by BEAP and these were higher than MIP and BCG respectively. Moreover, there was no mortality in any of the MEAP/BEAP immunized groups (Fig. 5b). The autopsy of dead animals in the control group confirmed a number of lesions in the lungs.

Further, the bacterial load at the end of 16 weeks post infection was highest in lung (Fig. 5c) and spleen (Fig. 5d) of control group. The lungs of MEAP immunized group had log (CFU) load of 3.68 which was 1.2 log (CFU) less than the MIP immunized group. Similarly, the log (CFU) load in BEAP immunized group was less than BCG immunized animals. Identical trend of CFU was observed in the spleen of all the groups. For all experiments, animals in each group were similar and no adverse event was observed. These findings reinforce that the alginate coated mycobacterium aerosol vaccines have enhanced protective potential.

#### Histo-pathological examination of mouse lungs infected with *M.tb*. H37Rv

**Fig. 6:**
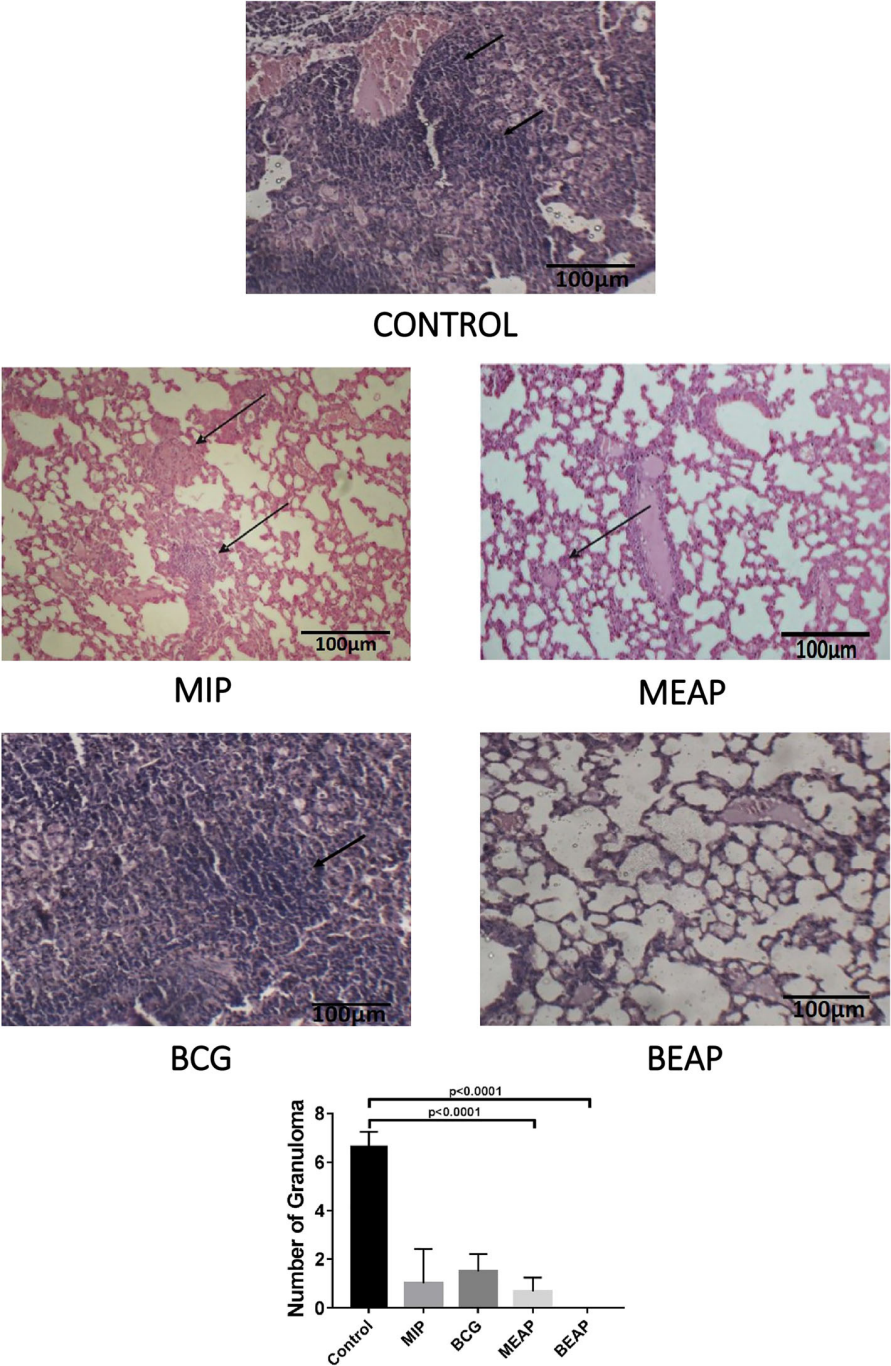
Histopathology of lungs from control, BCG, MIP, BEAP and MEAP immunized groups, post sixteen weeks of H37Rv infection. The arrows in figure indicate granulomatous lesions. The bar graph graph shows the summary of granulomatous lesions by randomly selecting 10 fields from 2 sections in each group

Sixteen weeks post infection, lung sections were examined after ‘blinding’ the source from the BCG, BEAP, MIP and MEAP immunized and control groups. There was less number of granulomatous lesions in the lung sections of BEAP/MEAP immunized mice. These lesions were well-defined and comprise majority of epithelioid and foamy cells as shown in Fig. 6. Some dense lymphocytic infiltrate was also present around small vessels. Lungs sections of mice immunized with BCG/MIP aerosol showed relatively diffuse infiltrate of granuloma with prominent perivascular lymphocytic infiltrates and a large number of lymphocytes throughout the parenchyma. In the control group severe necrotic pathology with no organized granulomatous lesions were seen. The perivascular spaces showed severe pneumonia with no focal points. These histological findings establish that the BEAP/MEAP immunized animals have reduced lung pathology as compared to the BCG/MIP immunized mice respectively.

## Discussion

The primary objective of this work was to encapsulate MIP and BCG into inhalable alginate particles of 2–4 μm size which could be formulated as dry powder aerosol (DPA) and to evaluate their immunogenic and protective efficacy in animal model of tuberculosis. We have demonstrated that interaction of DCs with alginate encapsulated MIP/BCG leads to significantly higher activation of DCs compared to the activation by aerosol of plain MIP/BCG. Similarly, the immune response and protective efficacy of BEAP and MEAP immunized animals were significantly higher than their respective liquid aerosols.

There have been a few reports on the preparation and delivery of mycobacterium DPA formulations. In these reports the DPA of BCG and *Mycobacterium smegmatis* was prepared by spray drying with iso-leucine [24, 25] and the DPA of bacteria was delivered directly to the lungs. Immediately after the delivery of these DPA formulations to the lungs, the plain mycobacterium was exposed to the micro-environment of alveoli and the cascade of immune reaction initiated.

In our approach, the micro-particles were prepared by encapsulating mycobacterium in the alginate. The alginate coating over the mycobacterium leads to many immunological advantages such as superior activation and maturation of DCs, which was also demonstrated by Yoshida et al. [26–29]. Additionally, there are few reports where activated DCs have been used with mycobacterial vaccines for better protective immunity against tuberculosis [30, 36–40].

The particle size distribution in an aerosol is very crucial for its efficient delivery to the lungs. Particles bigger than the 4 μm are not inhaled deep inside the lungs and most of the particles smaller than 2 μm are not retained in the lungs and are exhaled [41]. To achieve the crucial size range of 2–4 μm, the aggregates of particles in the lyophilized powder were segregated in an air jet mill [42, 43]. The jet milled preparations had necessary flow properties required by our delivery apparatus. The shelf life of jet milled formulation was more than 6 months (Fig. 1h) which is comparable to the shelf life of formulations reported by Wong et al. [25].

It is a well-known survival strategy of mycobacterium that after the phagocytosis, phagosome harboring mycobacterium inside is unable to fuse with lysosome. The confocal microscopy (Fig. 3a and c) pictures provide direct evidence for the uptake of MEAP/BEAP by DCs and their co-localization with lysosome indicating better processing of MEAP/BEAP and hence efficient antigen presentation to T cells in lymph node. These images also suggest that MEAP/BEAP were taken up more efficiently than MIP/BCG without any encapsulation. Similar findings have been reported by other investigators [27].

We investigated interaction of alginate coated mycobacterium formulations with DCs. After engulfing the antigen from the lung, DCs migrate to lymph node to presents the antigen to the T cells in lymph node. The up-regulation of CCR7 marker on the activated DCs is an indicator of their capacity of migration. It has also been reported that up-regulation of homing marker CCR7 on DCs make them more effective to present the antigen to T-cell [44]. In this study, we have shown that MEAP/BEAP activated BMDCs show higher up regulation of CCR7 receptor on their surface as compared to MIP/BCG activated BMDCs.

It has been demonstrated earlier [45] that the cellular immune response is not initiated in the lungs, though the lungs are the site of infection with *M. tuberculosis*. The initiation of the adaptive immune response requires transport of bacteria from the lungs to the mediastinal lymph node. After *M. tuberculosis* infection, the frequency of infected DCs increased in the lungs but their trafficking to the mediastinal lymph node is reduced; which ultimately result in a compromised initiation of naive CD4 T cell activation. This delay in T cell activation is believed to be responsible for the expansion of bacterial population in the lungs many folds [45] before the appearance of the adaptive immune response in the lungs.

In our experiments, the higher up regulation of CCR7 by MEAP/BEAP was translated into enhanced migration of BMDCs (Fig. 3e) in response to the chemotaxis induced by CCL21, which is an agonist for the chemokine receptor CCR7. As a result of this enhanced migration the adaptive immune response is mounted rather quickly and it confronts a smaller bacterial burden in the lungs.

In a similar experiment, Blomgran and Ernst [46] have demonstrated that DCs, those were directly infected by bacteria migrated poorly than DCs those had acquired the bacteria through uptake of infected neutrophils. In our experiments, the BCG/MIP encapsulated alginate particles have an analogy with infected neutrophils and they act like a delivery module of BCG/MIP to the DCs and this mode of delivery of bacteria to the DCs does not impair their mobility.

In addition to this the alginate encapsulation of live bacilli induce higher maturation of the BMDCs and lung DCs compared to the suspension of bacilli. This was indicated by the marked up-regulation of co-stimulatory molecules CD80, CD86 and MHC II (Table 1).

It is interesting to note that some activation has been observed with blank alginate particles (BAP) contrary to previously published reports where micro particles without strong antigens did not induce maturation of DCs [47]. The immuno-stimulatory capacity of DCs is not limited to the up-regulation of membrane bound co-stimulatory molecules but also depends on secreted soluble cytokines. We observed enhanced secretion of two important cytokines, IL-12 and TNF-alpha, from DCs on stimulation with MEAP/BEAP compared to MIP/BCG (Fig. 2a and b). Both the cytokines are considered to be modulator of T cell response towards Th1 type and a stronger Th1 response can limit the growth of mycobacterium [48].

Further, DCs stimulated by bacteria containing micro particles (MEAP/BEAP) show enhanced antigen presentation capacity as observed by high IFN-gamma release and high proliferation index when co-cultured with allogenic splenocytes. It has been proposed that DCs may exist in three developmental stages: immature, semi-mature and fully mature cells [49]. While immature and semi-mature DCs are implicated in the initiation of tolerance, only fully mature DCs can induce immunity. The higher amount of IFN-gamma in the allogenic MLR supernatant (Fig. 2) was a signature of superior maturation of DCs when incubated with MEAP/BEAP, which translated into better immunity against bacteria*.*

All these data imply that encapsulation of MIP/BCG into the alginate leads to the superior immune response against mycobacterium. When MEAP/BEAP were delivered to mice by our unique method the LDH assay confirms that it leads to no tissue damage or untoward reaction (Fig. 3f) in the animal. Additionally, minimal and comparable allergic response (Fig. 3g) was invoked by the liquid aerosol and DPA.

The in vivo immunological evaluation of different formulations showed similar trend as observed in vitro. Visibly enhanced memory response was observed in the mediastinal lymph node and spleen of mice immunized with MEAP/BEAP. The higher activation of DCs by MEAP/BEAP, the up-regulation of CCR7 and MHCII leads to better antigen presentation and T cells stimulation which is finally translated in to a robust memory response in these groups of animals. Further, the enhanced memory response in the BEAP/MEAP immunized group was able to protect these mice better than the BCG/MIP immunized group, when infected with *Mycobacterium tuberculosis* (H37Rv). Both CFU data (Fig. 5) and histopathological evaluation (Fig. 6) of lung support the superiority of immunization by the DPA of BEAP/MEAP over the immunization by the aerosol of BCG/MIP.

## Conclusions

In this study we have demonstrated a methodology to encapsulate live mycobacterium (MIP and BCG) as a dry powder inhale-able formulation which remains viable for over 6 months at 37 °C. A non-invasive procedure was developed to deliver this formulation to small animals. We have demonstrated that these inhale-able vaccines of live mycobacterium are more immunogenic as compared to the aerosol of bacilli and they provide better protection in mice when infected with H37Rv. The alginate coated DPA of BCG/MIP are very promising vaccine candidates for tuberculosis; they do not require the cold chain for transportation and storage, provide better protection than conventional intradermal or liquid aerosol and their delivery does not require the needle and a syringe.

## Additional file

Additional file 1: Supplementary Information. (DOC 801 kb)

## Data Availability

All data generated or analysed during this study are included in this published article and its supplementary information files.

## Abbreviations

Allo-MLR: Allogenic mixed lymphocyte reaction
BAP: Blank alginate particles
BCG: Bacille Calmette–Guérin
BEAP: BCG encapsulated alginate particles
BMDCs: Bone marrow dendritic cells
CFU: Colony forming units
DCs: Dendritic cells
DPA: Dry powder aerosol
ELISA: Enzyme linked immunosorbent assay
GM-CSF: Granulocyte monocyte colony stimulating factor
H37Rv: *Mycobacterium tuberculosis* H37Rv strain
IFN gamma: Interferon gamma
*M. tb*: *Mycobacterium tuberculosis*
MEAP: MIP encapsulated in alginate particles
MIP: *Mycobacterium indicus pranii*
