# Supplementary material for: Aerosol immunization by alginate coated mycobacterium (BCG/MIP) particles provide enhanced immune response and protective efficacy than aerosol of plain mycobacterium against M.tb. H37Rv infection in mice
Source: BMC Infect Dis. 2019 Jul 1;19:568. doi: 10.1186/s12879-019-4157-2 (PMC6604382; doi:10.1186/s12879-019-4157-2)
Supplement: Supplementary file 1 — Supplementary Information. (DOC 801 kb) [file 12879_2019_4157_MOESM1_ESM.doc]

**Supplementary Information:**

Materials and Methods

D(+) Trehalose dehydrate, Calcium chloride, Polyvinyl alcohol (M.W. 30,000 - 70,000) was purchased from Sigma. The sodium salt of Alginic acid (brown algae) was procured from Fluka Biochemika. For all experiments, MilliQ water was used. Cells for in vitro experiments were maintained in RPMI (Biological Sciences) supplemented with 10% v/v fetal bovine serum (FBS) (Biological Sciences) and 1% antibiotic solution procured from Sigma. All other tissue culture reagents and chemicals required for buffer were purchased from Sigma–Aldrich. *Mycobacterium* cultures were grown in either Difco Middlebrook 7H9 liquid or 7H11 solid Media (BD biosciences) supplemented with 10% ADC or OADC solution. For long term storage, cultures were maintained on LJ media (BD Bioscience). Hygromycin antibiotic required for GFP *Mycobacterium* was procured from Himedia. Danish 1331 strain Bacillus Calmette-Guérin (BCG) and an “in-house” maintained strain of “Mycobacterium indicus pranii” (MIP) were used. PE conjugated CD86 antibody was purchased from BD Biosciences and all other antibodies were acquired from e-bioscience.

*Animals*

All animal experiments were carried out on inbreed, 4-6 weeks old C57 BL/6 mice and BALB/c, obtained from Jacksons laboratory and bred and maintained in the small animal facility of the Institute. Animals were maintained according to the standard guidelines. All experimental procedures on animals were approved by the Institutional Animal Ethics Committee of National Institute of Immunology, New Delhi, India; certificate number IAEC/149/06 and IAEC/354/14.

*Preparation of viable Mycobacterium encapsulated alginate particles*

MIP/BCG cultures were gown in 7H9 media supplemented with 10% ADC in a shaking incubator at 150 rpm at 37°C. At optical density 0.9, culture was harvested by centrifugation at 1000 g for 10 minutes at 4°C. The bacterial pellet obtained was washed twice with phosphate buffer saline (PBS) pH 7.2. Pellet equivalent to 10^10^ bacilli was re-suspended in 50 ml solution containing 1.23% sodium alginate and 8.25% of trehalose. 5 ml of this suspension was filled in a laboratory made nebulization assembly and nebulized with a piston based air pump to generate aerosol of sodium alginate and MIP/BCG. A schematic diagram of nebulization assembly is shown below (Figure S1). The generated aerosol was entrapped in 5% solution of Calcium Chloride containing 0.1% polyvinyl alcohol with constant stirring. 12 h after the nebulization, gelled particles were collected by spinning at 350 g for 10 minutes. Particles were then washed three times with MilliQ water to completely remove residual Calcium chloride. Finally, the pellet was re-suspended in 5 ml MilliQ water, followed by snap freezing in liquid Nitrogen and dried in a lyophilizer.


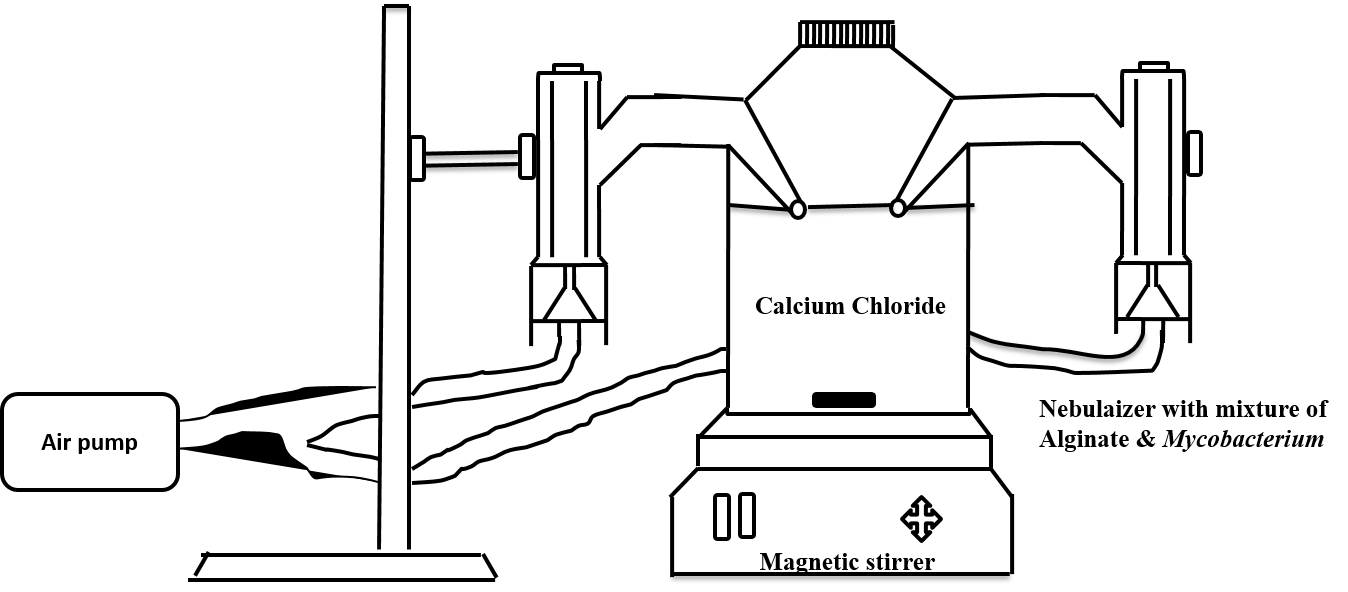


*Supplementary information, Figure S1 : Schematic diagram of the Nebulization assembly*

*Dissolution analysis*

After drying, around 2-4 mg weighed amount of the formulation was subjected to dissolution in 10 ml PBS to study the release profile of the bacilli from the particles. After predetermined time intervals 100 µl of the suspension was sampled and serial dilutions were plated on 7H11 agar supplemented with OADC. Plates were incubated at 37°C and the numbers of colony forming units (CFU) were counted after 2-3 weeks.

*Estimation of preload*

The initial load of MIP/BCG in the alginate particles was estimated by re-suspending 100µl of particle suspension, just before freeze-drying, in 10ml of PBS and kept for 8 h on shaker for particle dissolution. Different dilutions were then plated to estimate CFU load.

*JET milling for uniform sized formulation*

After lyophilization, alginate particles formed aggregates. They were segregated to individual particles by a high velocity air jet mill (Sturtevant®). For jet milling, lyophilized powder was fed via a hopper along with pressurized air at 100 psi feed pressure and grinded at 80 psi pressure. The jet-milled powder was stored in air tight tubes at 4°C till further use.

*Estimation of load in the particles before and after jet milling*

Weighed amounts of lyophilized and jet milled powders were re-suspended in 10 ml PBS and were kept over a rotor for 8 h at 37°C. Released bacilli were estimated as CFUs as described earlier.

*Scanning Electron Microscopy*

Shape and morphology of the micro particles was examined by scanning electron microscopy. Dried micro particles were attached to specimen stubs using double-sided tape and sputter-coated with gold–palladium in the presence of Argon. Micro particles were imaged with a CARL ZEISS EVO LS10 scanning electron microscope using a 22 kV accelerating voltage, a 10 mm working distance and 16mm objective aperture.

*Particle size analysis*

Size analysis of all the samples was done using Malvern size analyzer (MASTERSIZER 2000). 1-2 mg of dried formulation was re-suspended in ethanol and mixed in the suspension tank of size analyzer till 5% obscuration level and the size analysis was done using Mastersizer 2000 software.

*Dry Powder delivery directly to lungs of mice*

Formulation was delivered directly to the lungs of 6-8 weeks old C57BL/6 mice by endotracheal intubations. Mice was anesthetized by intra-peritoneal injection of 10μL Ketamine + 40μL Xylazine + 50μL saline solution and was lied down in ‘dorsal side up’ position on a 45° inclined platform specially made for this purpose. Trachea of the mice was viewed with an otoscope. A guiding wire with intubations tube was placed in the trachea by viewing through the otoscope. Once the tube was placed in the trachea guiding wire was removed from the tube. The dry powder formulation was aerosolized by using the apparatus shown below Figure S2, and 1mL aerosol was delivered to the lungs through intubation tube. After delivery of the formulation, the intubation tube was removed immediately and the mice were monitored till recovered from anesthesia.

**
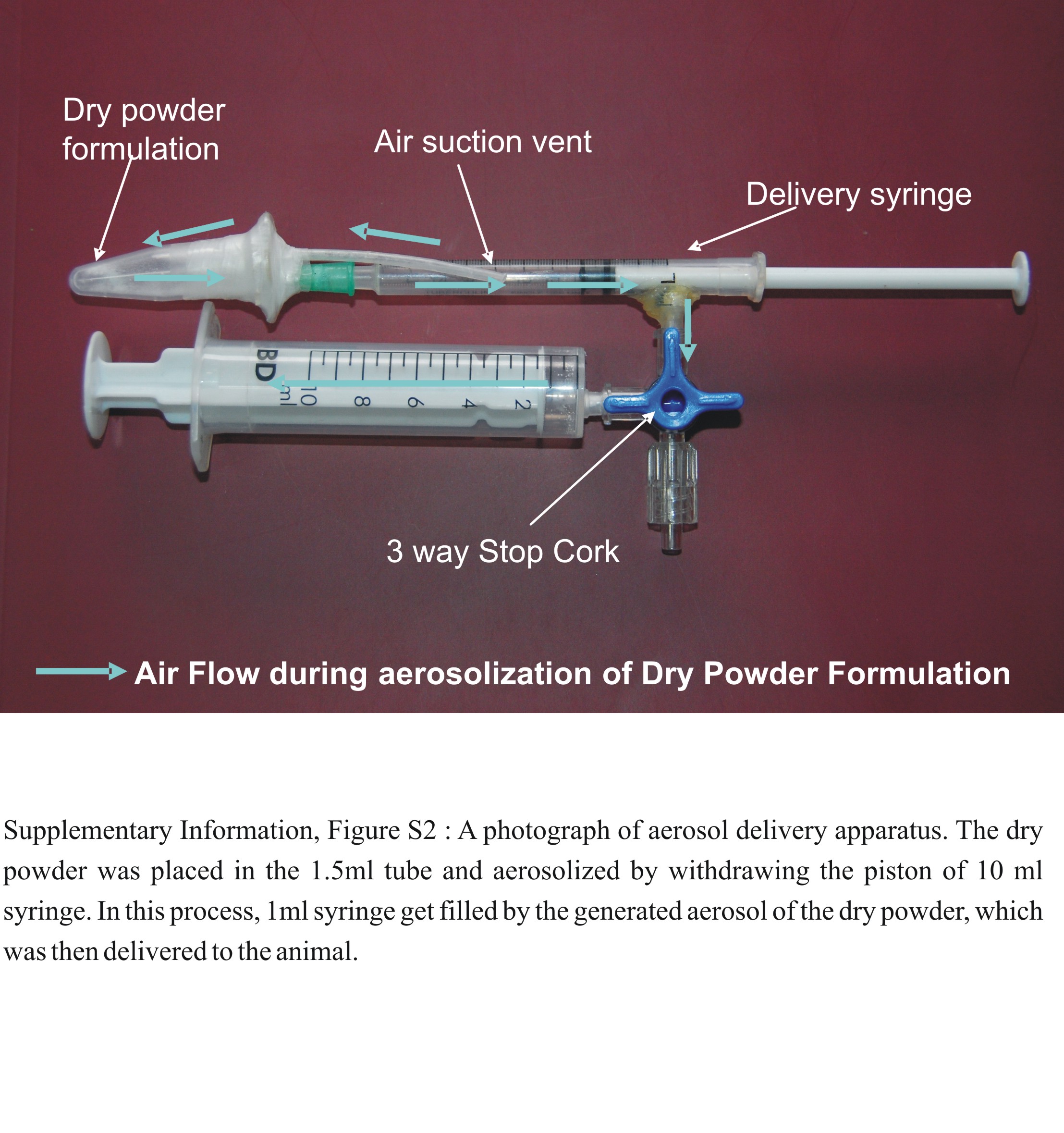
**

*Dose estimation in lung*

In order to estimate the dose delivered to the lungs, MEAP/BEAP was given to a group of mice. After 2 h, animal was sacrificed and both the lungs were homogenized in 1 ml of 7H9 media and bacilli load was estimated by plating.

*Bone marrow dendritic cell culture*

Bone marrow dendritic cells (BMDCs) were generated by in vitro culture of bone marrow cells from C57BL/6 mice in RPMI supplemented with 10ng/ml GM-CSF as per standard protocol [2]. On day 7, non adherent cells were harvested and used as immature DCs. 3 million cells per well were treated with 1.5 mg blank alginate particle (BAP) or 3 × 10^6^ bacilli as MEAP or BEAP or 3 × 10^7^ BCG or MIP. After 48 hrs of culture, the supernatant was collected and preserved for cytokine (TNF-α and IL-12) analysis by ELISA. Cells in the wells were then washed twice with PBS, harvested and stained for flow cytometry analysis.

*Staining for Flow Cytometry*

For flow cytometry staining, 5×10^5^/well BMDCs were incubated with antimouse CD16/32 antibodies in the staining buffer (PBS containing 0.1% sodium azide and 2% FCS) to block FcγIII/II receptor. Cells were then stained separately for CD11c-biotinylated followed by streptavidin FITC, CD86-PE, CD80-PE, MHCII-PE and CCR7 antibody for 45 minutes on ice. Labeled cells were washed and incubated with secondary antibody if required. Stained cells were fixed in 4% paraformaldehyde and stored at 4°C till analyzed. Samples were analyzed on a BD LSR flow cytometer and data analysis was done using WinMDI 2.8 shareware.

*Lung DC isolation*

A published procedure [3] was modified to isolate lung DCs. Briefly, lungs were flushed with 0.9% NaCl to remove all blood from the pulmonary circulation, and lungs dissected carefully to avoid all regional lymphoid tissues. Lungs were mechanically and enzymatically digested in a DNAase/collagenase solution at 35°C and lung mononuclear cells were isolated after passage over a Percoll density gradient (1.03/1.075) (Pharmacia). Anti-CD11c magnetic microbeads (Miltenyi Biotec) were used to isolate DCs from lung mononuclear cells.

*Allogenic mixed lymphocyte Reaction (MLR) Reaction*

5 × 10^4^ of BMDC or lung DCs per well were plated in a 96 well plate. They were stimulated with different antigen 50 μg blank alginate particle (BAP) or 1× 10^5^ bacilli as MEAP or BEAP or 5 × 10^5^ BCG or MIP. After 48 h of activation, DCs were co-cultured with 5 × 10^5^ allogenic splenocytes isolated from Balb/c mice for 48 h. After another 48 hrs, culture supernatant was collected from one set of wells for the cytokine analysis. In the remaining wells, after 60 hrs, 0.5 µ curie/well of tritiated thymidine was added. After 16-18 h of thymidine incorporation, cultures were harvested on printed filtermats (Wallac) by micro 96 harvester (Molecular Devices) and thymidine uptake was measured by β scintillation counter (PerkinElmer).

*ELISA*

Secreted IFN-gamma, TNF alpha and IL-12 were estimated by sandwich ELISA kits commercially available from e-biosciences or BD and suggested protocol followed.

*LDH and IgE estimation*

Two group of mice was given liquid aerosol of *Mycobacterium* and DPA of the *Mycobacterium* formulations as stated above. After 4, 24 and 48 h of delivery, bronchoalveolar lavage (BAL) fluid was collected from each treated group. From another set of mice, blood was sampled from recto-orbital plexus, serum separated and frozen at -20°C for later analysis. LDH and IgE levels were measured in BAL fluid and serum by commercially available kits. (LDH kit by EIAab & USCNLIFE and IgE kit by Immunology Consultants Laboratory).

*Transfection of BCG/MIP with GFP plasmid*

GFP clone of *BCG/*MIP was prepared by transfecting with vector pSC301 (obtained from Dr. Vinay Nandicoori, NII) as per standard protocol for *Mycobacterium* transfection[1]*.* Briefly, MIP/BCG culture was grown in 100 ml 7H9 media till OD 1 and processed for the preparation of electro competent cell (ECM). 200µl of ECM and 1µg of plasmid (approximately 2µl) were mixed and subjected to a pulse of 1.5 KV. After 3 h cells were harvested and plated on 7H11 agar media supplemented with 10% OADC and Hygromycin (100µg/ml) in different dilution in triplicate and incubated at 37°C till the colonies appear.

*Confocal Microscopy*

To microscopically visualize the uptake of particles by BMDCs, green fluorescent protein (gfp) expressing MIP or BCG or their corresponding MEAP/BEAP were used. In brief, 2×10^5^ DCs were seeded per well of 8 well chamber slides and stimulated with either of MEAP, BEAP, and MIP or BCG bacilli. After 48hrs, cells were washed with PBS and further incubated for 30 minutes with RPMI containing 20mM lysotracker DND 99 dye. After 30 minutes cells were washed three times with PBS and fixed in 4% paraformaldehyde-PBS. This was followed by staining with CD11c-Alexafluor 498 for 30 minutes. Slides were washed with PBS and fixed in Vector® mount with DAPI. Slides were examined in LCM Meta 5.10 Carl Zeiss Confocal Microscope and images were processed by AIM 4.2 software.

*Proliferation assay*

After 2 and 4 weeks of booster dose of immunization, spleens were removed from the mice and splenocytes were isolated. 2 × 10^5^ splenocytes per well were plated in two 96 well plates and stimulated with 2 µg per well of soluble *M.tb* or MIP antigen in triplicates. Plates were incubated at 37°C, 5% CO_2_ and after 48 hrs supernatants were harvested from wells of one of the plates for IFN-gamma estimation. In another plate, [^3^H] Thymidine (0.5 µ Curie per well) was added after another 12 hrs and further incubated for 16-18hrs and thymidine uptake measured.

List of References :

1. Goude R, Parish T. Electroporation of Mycobacteria. In: Methods in molecular biology (Clifton, N.J.). 2009. p. 203–15. doi:10.1007/978-1-59745-207-6_13.

2. Inaba K, Inaba M, Romani N, Aya H, Deguchi M, Ikehara S, et al. Generation of large numbers of dendritic cells from mouse bone marrow cultures supplemented with granulocyte/macrophage colony-stimulating factor. The Journal of experimental medicine. 1992;176:1693–702. http://www.ncbi.nlm.nih.gov/pubmed/1460426. Accessed 24 Jan 2019.

3. Thiele AT, Sumpter TL, Walker JA, Xu Q, Chang C-H, Bacallao RL, et al. Pulmonary Immunity to Viral Infection: Adenovirus Infection of Lung Dendritic Cells Renders T Cells Nonresponsive to Interleukin-2. Journal of Virology. 2006;80:1826–36. doi:10.1128/JVI.80.4.1826-1836.2006.
